# Current practices in physical fitness assessment and monitoring among coaches of individual and team sports: a survey in Portugal, Spain, and Romania

**DOI:** 10.5114/biolsport.2024.139074

**Published:** 2024-04-25

**Authors:** XiaoYuan Wen, Rui Miguel Silva, Qi Xu, Francisco Tomás González-Fernández, Ana Filipa Silva, Georgian Badicu, Xiaodan Guo, Filipe Manuel Clemente

**Affiliations:** 1 ChengDu Sports Univ, Chengdu 610041, Peoples R China; 2 Research Center in Sports Performance, Recreation, Innovation and Technology (SPRINT), 4960-320 Melgaço, Portugal; 3 Escola Superior Desporto e Lazer, Instituto Politécnico de Viana do Castelo, Rua Escola Industrial e Comercial de Nun’Álvares, 4900-347 Viana do Castelo, Portugal; 4 Gdansk University of Physical Education and Sport, 80-336 Gdańsk, Poland; 5 Department of Physical and Sports Education, Faculty of Education and Sports Sciences, Melilla Campus, University of Granada, 52006 Melilla, Spain; 6 Department of Physical Education and Special Motricity, Transilvania University of Brasov, 500068 Brasov, Romania

**Keywords:** Training monitoring, Recovery, Well-being, Athletic performance, Sports training

## Abstract

The objective of this study was to characterize surveyed coaches and elucidate the practices of physical fitness assessment and monitoring for both male and female athletes across three countries. A total of 165 coaches participated by completing a comprehensive 32-question survey. Pre-season assessments are a priority for coaches, with a significant range from 60.5% to 87.7% in Romania, while Portuguese and Spanish coaches tend to prefer testing during the competition (26.3% and 16.9%, respectively). Portuguese and Spanish coaches predominantly favor aerobic tests (50% and 46.8% respectively), whereas Romanian coaches exhibit a preference for sprint (56.9%) and skill tests (52.3%). Notably, change of direction tests are less commonly employed, ranging from 10.5% to 21% across the countries. In terms of exercise intensity determination, Portuguese coaches predominantly employ maximal heart rate (31.6%), while Spanish coaches often rely on the 220-age formula or perceived exertion (27.4%). For strength assessment, Portuguese coaches lean towards direct (34.2%) or estimated (31.6%) maximal repetition methods. When it comes to maximal speed sprint, Portuguese and Romanian coaches show preference (50% and 43.1% respectively), while Spanish coaches exhibit a relative lack of emphasis on individualized speed measures (37.1%). Perceptual scales are the preferred method for recovery monitoring, with adoption rates of 57.9% in Portugal, 53.2% in Spain, and 44.6% in Romania. In summary, this study underscores the distinct assessment and monitoring practices employed by coaches in Portugal, Spain, and Romania. These findings are in alignment with established literature standards, highlighting the diversity of approaches used in different countries.

## INTRODUCTION

The implementation of periodic assessments and monitoring practices allows coaches and practitioners to evaluate athletes’ overall physical fitness status, and how they are coping with the training process [1]. The periodic assessments focus on quantifying and qualifying the athletes’ anthropometric characteristics, body composition, physical qualities, technical capacities, and tactical behaviors. On the other hand, monitoring allows for a comprehensive picture regarding the responses to the imposed external training loads, such as objective and subjective measures of training intensity, well-being, and readiness [2]. By conducting periodic assessments and monitoring, coaches can identify immediate and long-term responses to training, adjusting training according to the athlete’s individual needs.

Different laboratory- and field-based tests are available to assess the physical fitness status of athletes [3, 4]. However, in the overall sports setting, implementing laboratory tests can be challenging due to logistical and time constraints. Therefore, previous studies have recommended the use of a field-based testing battery specifically designed for youth and adult sports contexts [5, 6]. Before selecting which tests to include in the battery, a needs analysis should be conducted to determine the specific physical and physiological demands of the sport. Moreover, conducting physical fitness tests at a single time point is insufficient for providing a comprehensive evaluation of an athlete’s overall fitness levels [6]. To comprehensively understand an athlete’s physical capabilities, it’s crucial to regularly conduct periodic fitness assessments [3].

Training monitoring practices play a crucial role in optimizing athlete performance and minimizing the risk of injuries. Understanding sports training load involves breaking it down into two main components: internal and external sports training load [7]. External sports load encompasses measurable training demands like distance covered, sprint distance, and accelerations [8]. Conversely, internal sports training load refers to physiological and psychological responses, including heart rate, perceived exertion, well-being, and readiness [9]. Coaches must also consider individual differences, such as fitness level, age, sex, and injury history, to tailor training programs effectively [10].

In soccer, monitoring practices are particularly prevalent, aiming to track player training load, recovery, and injury risk [11]. Various technologies, including GPS, heart rate monitors, and accelerometers, are utilized to measure different aspects of player performance and health [12]. A recent study delved into the training load monitoring practices and perceptions of coaches and practitioners in elite soccer [13]. Surveying 118 participants, the above-mentioned study found a general consensus on the importance of training load monitoring, despite some discrepancies in practices and perceptions. Coaches often relied on subjective measures like RPE, while practitioners preferred objective measures such as GPS and heart rate monitoring [13]. Additionally, a comprehensive review investigated training monitoring methods across sports, revealing a lack of consensus among coaches and practitioners who employed a wide range of approaches [14].

While some studies have explored strength and conditioning coaches’ methodologies in various sports [15–17], these primarily characterize common practices without delving into specific monitoring approaches. Notably, existing research on training monitoring practices is limited, with a predominant focus on soccer [18]. Other studies aimed at developing monitoring guidelines lack a comprehensive survey of practices and needs across diverse sports [19]. Only one systematic review has investigated monitoring practices, but it included studies of different designs without coach surveys [14]. Similarly, only one study, confined to football, surveyed coaches on periodic assessment practices [13]. The present study addresses these critical gaps by providing a comprehensive survey-based exploration of periodic assessment and monitoring practices across diverse sports.

Given that, the present study aimed to survey several coaches from a broad range of both individual and collective sports with certified credentials from their respective sports federations, to: (i) characterize the population of surveyed coaches; (ii) describe the implemented periodic assessments; and (iii) describe the implemented monitoring by sports coaches of male and female athletes from different sports.

## MATERIALS AND METHODS

### Study design

This study employed a descriptive cross-sectional research design to investigate and provide comprehensive insights into the prevailing practices of sports coaches concerning the implementation of periodic assessments and training monitoring procedures. Participants, spanning various coaching roles (head coaches, assistant coaches, sports scientists, strength and conditioning coaches, and physical trainers), were recruited from three distinct countries, representing diverse sports disciplines. The study aimed to gather detailed insights into how coaches currently employ periodic assessments and training monitoring in different sports contexts, with a focus on understanding predominant trends and methodologies.

### Setting

A survey was conducted online using Google Forms and distributed via email to all the coaches participating in this study. Coaches of any gender, age group, or academic level were considered eligible for inclusion. There were no limitations to the fact that they currently exercise functions. Any sports coach of any nationality was considered eligible for the study, as long as they held a professional license validated by their respective sport’s official federation. The present study followed the ethical recommendations for the study in humans as suggested by the Declaration of Helsinki (updated version from 2013).

### Participants

A total of 165 coaches participated in the present study. 38 coaches from Portugal, 62 from Spain, and 65 from Romania responded to the survey. All coaches provided informed consent to initiate the anonymous online survey, and only fully completed surveys were used for analyses. Also, the anonymity and confidentiality of your data will be ensured and maintained, and it is an obligation to guarantee confidentiality and the researchers’ duty of secrecy towards the subject evaluated. An anonymous report was produced to provide global information on the evaluation processes and training control carried out by all participating sports coaches, and the participants could request the reports. The participation was completely voluntary, and if at any time during the course of the study any participant considered that any intervention/question put them in an uncomfortable situation, they could leave the questionnaire without having to justify themselves. The study received approval from the Ethics Committee for Social Sciences, Life, and Health at Instituto Politécnico de Viana do Castelo, under reference number 4/A/2023.

Table 1 provides an overview of the participants’ characteristics and backgrounds, including their academic qualifications, certificate levels in sports coaching, experience, and the competitive levels in which they are currently involved.

**TABLE 1:** Demographic characterization of survey respondents.

	Portuguese	Portuguese	Spanish	Spanish	Romanian	Romanian

	Men (N = 31)	Women (N = 7)	Men (N = 55)	Women (N = 7)	Men (N = 41)	Women (N = 9)
**Age**						
18–25 years old	1 (3.2%)	0 (0%)	16 (29.1%)	1 (14.3%)	0 (0%)	4 (16.7%)
26–30 years old	1 (3.2%)	1 (14.3%)	10 (18.2%)	1 (14.3%)	3 (7.3%)	1 (4.2%)
31–35 years old	2 (6.5%)	0 (0%)	6 (10.9%)	4 (57.1%)	5 (12.2%)	0 (0%)
36–40 years old	4 (12.9%)	0 (0%)	6 (10.9%)	0 (0%)	9 (22.0%)	2 (8.3%)
41–45 years old	5 (16.1%)	2 (28%)	5 (9.1%)	0 (0%)	8 (19.5%)	8 (33.3%)
46–50 years old	4 (12.9%)	0 (0%)	5 (9.1%)	0 (0%)	3 (7.3%)	3 (12.5%)
51–55 years old	4 (12.9%)	3 (42.9%)	6 (10.9%)	0 (0%)	5 (12.2%)	5 (20.8%)
> 55 years old	10 (32.3%)	1 (14.3%)	1 (1.8%)	1 (14.3%)	8 (19.5%)	1 (4.2%)

**Education**						
Basic education	0 (0%)	0 (0%)	2 (3.6%)	0 (0%)	0 (0%)	0 (0%)
Secondary school	10 (32.3%)	0 (0%)	8 (14.5%)	0 (0%)	2 (4.9%)	0 (0%)
Graduation	14 (45.2%)	3 (42.9%)	34 (61.8%)	3 (42.9%)	13 (31.7%)	8 (33.3%)
Master degree	6 (19.4%)	2 (28.6%)	7 (12.7%)	2 (28.6%)	23 (56.1%)	14 (58.3%)
Ph.D. degree	1 (3.2%)	2 (28.6%)	4 (7.3%)	2 (28.6%)	3 (7.3%)	2 (8.3%)

**Role in Sports**						
Head coach	28 (90.3%)	3 (42.9%)	38 (69.1%)	5 (71.4%)	33 (80.5%)	15 (62.5%)
Assistant coach	1 (3.2%)	1 (14.3%)	5 (9.1%)	0 (0%)	0 (0%)	3 (12.5%)
Physical trainer	1 (3.2%)	0 (0%)	5 (9.1%)	1 (14.3%)	1 (2.4%)	0 (0%)
Physiologist	1 (3.2%)	1 (14.3%)	0 (0%)	0 (0%)	0 (0%)	0 (0%)
Match analyst	0 (0%)	0 (0%)	1 (1.8%)	0 (0%)	0 (0%)	0 (0%)
Other	0 (0%)	2 (28.6%)	6 (10.9%)	1 (14.3%)	7 (17.1%)	6 (25.0%)

**License Level**						
Without level	0 (0%)	9 (0%)	9 (16.4%)	1 (14.3%)	0 (0%)	0 (0%)
Level I	4 (12.9%)	3 (42.9%)	20 (36.4%)	0 (0%)	19 (46.3%)	7 (29.2%)
Level II	11 (35.5%)	3 (42.9%)	11 (20.0%)	4 (57.1%)	11 (26.8%)	2 (8.3%)
Level III	14 (45.2%)	1 (14.3%)	15 (27.3%)	2 (28.6%)	6 (14.6%)	10 (41.7%)
Level IV	2 (6.5%)	0 (0%)	0 (0%)	0 (0%)	5 (12.2%)	5 (20.8%)

**Experience in Coaching**						
0–5 years of experience	8 (25.8%)	4 (57.1%)	28 (50.9%)	2 (28.6%)	9 (22.0%)	10 (41.7%)
6–10 years of experience	2 (6.5%)	2 (28.6%)	14 (25.5%)	4 (57.1%)	8 (19.5%)	4 (16.7%)
11–15 years of experience	5 (16.1%)	0 (0%)	4 (7.3%)	1 (14.3%)	4 (9.8%)	4 (16.7%)
16–20 years of experience	4 (12.9%)	1 (14.3%)	8 (14.5%)	0 (0%)	4 (9.8%)	3 (12.5%)
21–25 years of experience	5 (16.1%)	0 (0%)	0 (0%)	0 (0%)	5 (12.2%)	2 (8.3%)
> 25 years of experience	7 (22.6%)	0 (0%)	1 (1.8%)	0 (0%)	11 (26.8%)	1 (4.2%)

Table 2 provides an overview of the different sports the surveyed coaches participate in.

**TABLE 2:** Sports of the surveyed coaches.

Sports	Portuguese (n = 38)	Spanish (n = 62)	Romanian (n = 65)
Handball	8 (21.1%)	0 (0%)	11 (16.9%)
Canoeing	6 (15.8%)	0 (0%)	0 (0%)
Gymnastics	0 (0%)	1 (1.6%)	5 (7.7%)
Golf	1 (2.6%)	0 (0%)	0 (0%)
Judo	2 (5.3%)	0 (0%)	4 (6.2%)
Swimming	2 (5.3%)	0 (0%)	5 (7.7%)
Skating	0 (0%)	0 (0%)	3 (4.6%)
Tennis	0 (0%)	0 (0%)	2 (3.1%)
Basketball	2 (5.3%)	1 (1.6%)	6 (9.2%)
Motorsports	0 (0%)	0 (0%)	1 (1.5%)
Climbing	0 (0%)	0 (0%)	4 (6.2%)
Futsal	1 (2.6%)	1 (1.6%)	0 (0%)
Orientation	0 (0%)	0 (0%)	1 (1.5%)
Rowing	2 (5.3%)	0 (0%)	0 (0%)
Table tennis	1 (2.6%)	0 (0%)	0 (0%)
Chess	0 (0%)	0 (0%)	1 (1.5%)
Athletics	2 (5.3%)	1 (1.6%)	2 (3.1%)
Adapted sports	1 (2.6%)	0 (0%)	0 (0%)
Fencing	0 (0%)	0 (0%)	2 (3.1%)
Rink hockey	1 (2.6%)	0 (0%)	0 (0%)
Paddle tennis	0 (0%)	1 (1.6%)	0 (0%)
Martial arts	0 (0%)	0 (0%)	3 (4.6%)
Archery	2 (5.3%)	0 (0%)	0 (0%)
Soccer	4 (10.5%)	57 (91.9%)	14 (21.5%)
Volleyball	3 (7.9%)	0 (0%)	1 (1.5%)

### Periodic assessments and monitoring survey

A survey was developed exclusively for this research project. Initially, the key domains and questions about periodic assessments and monitoring were formulated. This process involved collaborative efforts from three researchers, who based the questions on existing literature related to the subject [6, 14, 20–23]. To ensure the survey’s quality and validity, it underwent evaluation by three independent academic experts in periodic assessments and monitoring, all of whom were highly esteemed researchers according to ExpertScape. These experts had published a minimum of three recent papers on the subject. Their valuable feedback and comments on the wording and structure of the questions were thoughtfully considered, leading to revisions and reorganization of the survey. The main recommendations from experts emphasized the inclusion of specific practical scenarios clarifying the context in which monitoring processes took place.

Following these modifications, the refined survey was then distributed to three additional experts, all of whom were experienced coaches holding a minimum level II coaching license and possessing an academic background in the field. Their input and feedback played a vital role in further refining the survey. After incorporating the valuable suggestions from both the academic experts and coaches, the final version of the survey was once again sent to the initial three independent academic experts for their evaluation. Upon receiving their approval, the definitive survey, presented as a supplementary file, was launched online through Google Forms.

Before proceeding with the survey, participants were provided with a clear explanation of its aim, research design, and assurances concerning anonymity, confidentiality, and data protection. Participants voluntarily confirmed their willingness to participate and provided their consent to proceed with the questionnaire. The survey consisted of 32 closed-ended questions (no scales used). Five sections were included as follows (see supplementary material): (i) study information; (ii) personal and academic information; (iii) coaching experience; (iv) implementation of periodic assessments; (v) implementation of monitoring practices. An investment of 15 minutes was estimated to complete the questionnaire.

### Data acquisition and statistical procedures

The data acquired from the Google Forms survey was exported and consolidated into an Excel file for subsequent analysis. As all the questions required mandatory responses, there were no missing cases, and consequently, all the items were deemed valid upon inspection. Closed-ended questions were subjected to a frequency analysis, chosen for their ability to provide a systematic and comprehensive examination of the distribution and occurrence of various response options. This quantitative assessment, through frequency analysis, offers a clear understanding of the participants’ choices and preferences within the predefined response categories, allowing for a straightforward interpretation of the data. Frequency analysis was deemed particularly suitable for capturing the prevalence of specific responses, a key aspect in exploring the prevailing trends and patterns in coaches’ practices.

## RESULTS

Table 3 describes the characterization of the application of physical fitness, and technical and tactical periodic assessments. Based on survey results, player assessments are most frequently conducted during pre-season, ranging from 60.5% in Portugal to 87.7% in Romania. In Portugal, only 26.3% of coaches assess players during the season, while in Romania and Spain, the percentages are 16.9% and 45.2%, respectively. A noteworthy observation is that a small percentage of coaches in Portugal and Spain (13.2% and 14.5%, respectively) reported never assessing players, and in Romania, the percentage was even lower at 4.6%.

**TABLE 3:** Characterization of the application of physical fitness, technical and tactical periodic assessments.

	Portuguese (n = 38)	Spanish (n = 62)	Romanian (n = 65)
***When do you apply physical, technical, and/or tactical assessment tests?***			
During the pre-season	23 (60.5%)	46 (74.2%)	57 (87.7%)
During the competitive season	10 (26.3%)	28 (45.2%)	11 (16.9%)
After the season	0 (0%)	12 (19.4%)	7 (10.8%)
Never	5 (13.2%)	9 (14.5%)	3 (4.6%)

***How many times do you apply physical, technical, and/or tactical tests during the sports season?***			
0	6 (15.8%)	8 (12.9%)	12 (18.5%)
1	3 (7.9%)	7 (11.3%)	9 (13.8%)
2	8 (21.1%)	10 (16.1%)	19 (29.2%)
3	11 (28.9%)	22 (35.5%)	7 (10.8%)
4	2 (5.3%)	3 (4.8%)	11 (16.9%)
5	3 (7.9%)	6 (9.7%)	0 (0%)
6	2 (5.3%)	0 (0%)	1 (1.5%)
7	3 (7.9%)	0 (0%)	1 (1.5%)
8	0 (0%)	1 (1.6%)	1 (1.5%)
> 9	3 (7.9%)	5 (9.1%)	4 (6.2%)
All the sessions			

***What is the frequency with which you apply physical, technical, and/or tactical tests during the sports season?***			
Never	7 (18.4%)	10 (16.1%)	6 (9.2%)
Monthly	5 (13.2%)	10 (16.1%)	26 (40.0%)
Every two months	6 (15.8%)	10 (16.1%)	8 (12.3%)
Every three months	13 (34.2%)	18 (29.0%)	8 (12.3%)
Every four months	1 (2.6%)	8 (12.9%)	1 (1.5%)
Every five months	1 (2.6%)	2 (3.2%)	2 (3.1%)
Every six months	5 (13.2%)	4 (6.5%)	14 (21.5%)

***Which of the following tests do you use in the implementation of periodic assessment for your athletes?***			
None	6 (15.8%)	11 (17.7%)	1 (1.5%)
Anthropometry and body composition	17 (44.7%)	12 (19.4%)	21 (32.3%)
Maximum strength and power	19 (50.0%)	20 (32.3%)	20 (30.8%)
Muscular endurance	12 (31.6%)	18 (29.0%)	11 (16.9%)
Aerobic	19 (50.0%)	29 (46.8%)	26 (40%)
Anaerobic	13 (34.2%)	22 (35.5%)	19 (29.2%)
Speed and/or acceleration	15 (39.5%)	25 (40.3%)	37 (56.9%)
Change of direction	4 (10.5%)	13 (21.0%)	12 (18.5%)
Agility	7 (18.4%)	12 (19.4%)	17 (26.2%)
Mobility, stability, and/or flexibility	9 (23.7%)	14 (22.6%)	14 (21.5%)
Motor coordination	8 (21.1%)	13 (21.0%)	20 (30.8%)
Specific technical skills	16 (42.1%)	19 (30.6%)	34 (52.3%)
Specific tactical behaviors	5 (13.2%)	14 (22.6%)	17 (26.2%)

The majority of responders in Portugal and Spain reported assessing players three times a year, accounting for 28.9% and 35.5%, respectively. Conversely, in Romania, the majority reported assessing players twice a year, constituting 29.2%.

The frequency of player assessments varied among responders across countries. In Romania, most responders reported monthly testing, while in Portugal and Spain, the majority indicated assessments every three months, with percentages of 34.2% in Portugal and 29% in Spain.

Differences in the types of tests used by coaches for measuring athlete performance emerged across countries. In Portugal and Spain, the majority preferred tests for measuring aerobic performance, with percentages of 50% and 46.8%, respectively. In Romania, responders were more focused on Sprint tests (56.9%) and skill tests (52.3%). Notably, change-of-direction tests were the least employed across all three countries, with percentages varying from 10.5% in Portugal to 21% in Spain and 18.5% in Romania.

Table 4 characterizes the implementation by coaches of the most common measures used in practice. In Portugal, 31.6% of responders favored using maximal heart rate in field-based tests, while in Spain, the majority preferred the formula 220-age or the rate of perceived exertion (27.4%). In Romania, 26.9% reported utilizing training heart rate (maximal heart rate minus recovery heart rate) to estimate exercise intensity.

**TABLE 4:** Characterization of the periodic assessment measures used for practice.

	Portuguese (n = 38)	Spanish (n = 62)	Romanian (n = 65)
***How do you determine the intensity in the context of aerobic/anaerobic training?***			
I don’t determine	8 (21.1%)	18 (29%)	13 (20%)
Based on maximum heart rate	12 (31.6%)	8 (12.9%)	16 (24.6%)
Based on estimated maximum heart rate (e.g., 220-age)	10 (26.3%)	17 (27.4%)	16 (24.6%)
Based on training heart rate (considering both maximum and resting heart rates)	8 (21.1%)	11 (17.7%)	24 (36.9%)
Based on maximum oxygen volume measured through spirometry	2 (5.3%)	1 (1.6%)	2 (3.1%)
Based on estimated maximum oxygen volume in field tests	3 (7.9%)	0 (0%)	2 (3.1%)
Based on blood lactate levels	5 (13.2%)	4 (6.5%)	5 (7.7%)
Based on subjective perception of effort	7 (18.4%)	17 (27.4%)	5 (7.7%)
Based on maximum aerobic speed (measured with spirometry)	2 (5.3%)	0 (0%)	2 (3.1%)
Based on estimated maximum aerobic speed in field tests	3 (7.9%)	4 (6.5%)	7 (10.8%)
Based on anaerobic speed reserve	3 (7.9%)	1 (1.6%)	1 (1.5%)

***How do you determine intensity in strength training?***			
I don’t determine	9 (23.7%)	24 (38.7%)	23 (35.4%)
Estimation of one-repetition maximum (indirect method)	12 (31.6%)	9 (14.5%)	13 (20%)
One-repetition maximum	13 (34.2%)	9 (14.5%)	14 (21.5%)
Determination of the force-velocity curve	8 (21.1%)	8 (12.9%)	10 (15.4%)
Utilization of perceived methods (e.g., perceived exertion, repetitions in reserve)	5 (13.2%)	13 (21%)	7 (10.8%)
Training to failure	4 (10.5%)	5 (8.1%)	3 (4.6%)
Trial and error	3 (7.9%)	3 (4.8%)	0 (0%)

***How do you determine intensity in the context of speed training?***			
I don’t determine	17 (44.7%)	23 (37.1%)	11 (16.9%)
Based on maximum running speed	19 (50%)	19 (30.6%)	28 (43.1%)
Based on strength-speed profile	6 (15.8%)	22 (35.5%)	28 (43.1%)

***Do you use the assessment results in your training programming practice to direct the focus of training?***			
No	4 (10.5%)	15 (24.2%)	8 (12.3%)
Yes, in the case of physical qualities	24 (63.2%)	35 (56.5%)	44 (67.7%)
Yes, in the case of technical skills	19 (50%)	22 (35.5%)	31 (47.7%)
Yes, in the case of tactical behaviors	6 (15.8%)	22 (35.5%)	20 (30.8%)

***If you do not use assessments as markers for training programming, this is due to:***			
Not all tests are capable of providing information to adjust training	13 (34.2%)	29 (46.8%)	15 (23.1%)
Technically, I am unable to interpret the obtained values and convert them for programming	6 (15.8%)	15 (24.2%)	14 (21.5%)
I only use the data to assess the athletes’ condition	19 (50%)	18 (29%)	36 (55.4%)

Differences in methods for standardizing the intensity of strength training were evident. In Portugal, responders predominantly used direct maximal repetition (34.2%) or estimated maximal repetition (31.6%). In Spain and Romania, the majority reported not using any measure for standardization, with percentages of 38.7% in Spain and 35.4% in Romania.

Regarding speed training, in Portugal, 50% of responders reported using maximal speed sprint for standardization, while in Romania, it was either maximal speed sprint (43.1%) or force-velocity profile (43.1%). In Spain, 37.1% reported not using any measure for individualizing speed training.

Across all countries, a majority of responders (63.2% in Portugal, 56.5% in Spain, and 67.7% in Romania) used physical fitness test results to plan training. However, disparities emerged in the use of tactical behaviors. In Portugal, only 15.8% used tactical behaviors for planning, while in Spain and Romania, higher percentages of responders reported using tactical behaviors, with percentages of 35.5% in Spain and 30.8% in Romania.

Reasons for not using player assessments to individualize training varied. In Portugal and Romania, the major reason (50% and 55.4%, respectively) was using assessments solely for player status. In Spain, 46.8% reported not using assessments because not all could be applied to individualize training. Additionally, 15.8% in Portugal and 24.2% in Spain cited a lack of technical knowledge to interpret assessment values, emphasizing the need for coach education and development in interpreting and applying assessment results to individualize training programs.

Table 5 describes the implementation monitoring practices of coaches. In Spain, a majority (59.7%) reported no use of these monitoring tools in either training or matches. Conversely, in Portugal and Romania, the majority reported using these tools exclusively in training sessions, with percentages of 39.5% in Portugal and 52.3% in Romania.

**TABLE 5:** Characterization of the monitoring practices.

	Portuguese (n = 38)	Spanish (n = 62)	Romanian (n = 65)
***When do you apply effort monitoring systems (e.g., RPE, HR, GPS) and/or well-being (e.g., sleep quality, DOMS, stress, fatigue)?***			
Never	14 (36.8%)	37 (59.7%)	17 (26.2%)
On training days	15 (39.5%)	16 (25.8%)	34 (52.3%)
On match/game days	1 (2.6%)	6 (9.7%)	9 (13.8%)
Every day of the week	8 (21.1%)	5 (8.1%)	10 (15.4%)

***What instruments do you use to analyze effort during exercise/training?***			
I do not use	11 (29.8%)	31 (50.0%)	20 (30.8%)
Subjective perception of effort scales	10 (26.3%)	13 (21.0%)	8 (12.3%)
GPS and/or IMU	4 (10.5%)	8 (12.9%)	7 (10.8%)
Heart rate monitors	11 (28.9%)	10 (16.1%)	21 (32.2%)
Gas analyzer (spirometry)	0 (0%)	0 (0%)	0 (0%)
Lactate analyzer	1 (2.6%)	0 (0%)	3 (4.6%)
Kinematic systems (multiple cameras; smartphone)	1 (2.6%)	0 (0%)	6 (9.2%)

***What instruments do you use to analyze the technical/tactical performance in exercise/training?***			
I do not use	11 (28.9%)	18 (29.0%)	13 (20.0%)
Kinematic analysis systems (e.g., cameras)	11 (28.9%)	7 (11.3%)	25 (38.5%)
Observational and notational systems	12 (31.6%)	35 (56.5%)	24 (36.9%)
GPS (Global Positioning System) systems	4 (10.5%)	2 (3.2%)	3 (4.6%)

***What instruments do you use to analyze effort during competition?***			
I do not use	15 (39.5%)	28 (45.2%)	29 (44.6%)
Subjective perception of effort scales	12 (31.6%)	16 (25.8%)	7 (10.8%)
GPS and/or IMU	6 (15.8%)	9 (14.5%)	5 (7.7%)
Heart rate monitors	4 (10.5%)	7 (11.3%)	13 (20.0%)
Gas analyzer (spirometry)	0 (0%)	0 (0%)	0 (0%)
Lactate analyzer	0 (0%)	0 (0%)	4 (6.2%)
Kinematic systems (multiple cameras)	1 (2.6%)	2 (3.2%)	7 (10.8%)

***What instruments do you use to analyze technical/tactical performance during competitions?***			
I do not use	9 (23.7%)	20 (32.3%)	13 (20%)
Kinematic systems (multiple cameras)	11 (28.9%)	12 (19.4%)	31 (47.7%)
Observational and notational systems	15 (39.5%)	33 (53.2%)	29 (44.6%)
GPS systems	3 (7.9%)	4 (6.5%)	2 (3.1%)

***What instruments/measures do you use to analyze recovery?***			
Heart rate variability with heart rate monitors	12 (31.6%)	26 (41.9%)	22 (33.8%)
Blood markers	1 (2.6%)	0 (0%)	1 (1.5%)
Submaximal tests	1 (2.6%)	0 (0%)	3 (4.6%)
Saliva analysis	0 (0%)	0 (0%)	1 (1.5%)
Neuromuscular tests	2 (5.3%)	3 (4.8%)	9 (13.8%)
Well-being questionnaires	22 (57.9%)	33 (53.2%)	29 (44.6%)

RPE: rate of perceived exertion; HR: heart rate; GPS: global positioning systems; IMU: inertial measurement unit; DOMS: delayed onset muscle soreness

Differences in monitoring tools for controlling training intensity were evident. In Spain, 50% of responders reported no use of any monitoring tools for controlling training intensity. In Portugal and Romania, the majority used heart rate, with percentages of 28.9% and 32.3%, respectively. Both Portugal and Spain reported using rate of perceived exertion, with percentages of 26.3% in Portugal and 21% in Spain, while in Romania, only 12.3% of responders used these scales. These findings indicate a lack of consistency in the use of monitoring tools for controlling training intensity among coaches across countries and contexts.

Variations were observed in the instruments used for measuring technical/tactical behavior in training. In Spain, 56.5% reported using observational tools, while in Portugal, the majority also reported using observational tools (31.6%). In Romania, the majority used kinematic analysis (38.5%), with a significant implementation in Portugal (28.9%). However, only 11.3% of responders in Spain reported using kinematic analysis.

Differences in the use of instruments for measuring intensity in competition were noted. The majority of coaches in Portugal, Spain, and Romania reported not using instruments for measuring intensity in competition (39.5%, 45.2%, and 44.6%, respectively). Among those using such instruments, perceptive scales were predominant in Portugal and Spain (31.6% and 25.8%, respectively), while in Romania, 20% used heart rate monitors.

Similarities and differences emerged in the use of monitoring tools for technical/tactical performance. Observational analysis was the primary tool in all three countries, with percentages of 39.5% in Portugal, 53.2% in Spain, and 44.6% in Romania. In Romania, a significant percentage (47.7%) reported using kinematic analysis in competition, while in Portugal, 28.9% used it, and in Spain, only 19.4% reported its use.

Similarities and differences were also observed in the use of instruments/measures for monitoring recovery status. The majority in Portugal (57.9%), Spain (53.2%), and Romania (44.6%) used well-being perceptive scales. Additionally, in Portugal and Romania, the second most used instrument/measure was heart rate variability, with percentages of 31.6% and 33.8%, respectively. In Spain, a higher percentage (41.9%) of responders used heart rate variability.

## DISCUSSION

The aim of this study was to outline the methods of periodic assessments and monitoring that sports coaches employ across various sports in European countries. Among the participating coaches from the three nations, a predominant male representation was observed, with Spain exhibiting the highest male demographic. A majority of coaches held a bachelor’s degree, with a subset possessing master’s degrees, particularly evident in Romania. Principal roles within coaching were led by head coaches across all nations, while ancillary positions such as physiologists and match analysts exhibited modest engagement. Coaching tenure was primarily within the 0–5 year range, except in Portugal where some coaches demonstrated greater experience.

Notably, the evaluation of players predominantly occurred during the pre-season phase, although variations existed among the surveyed countries. Spain and Portugal favored maximal heart rate assessments, while Romania demonstrated a preference for sprint and skill evaluations. Importantly, outcomes of physical fitness tests played a pivotal role in the formulation of training programs. The incorporation of monitoring tools, encompassing training load and well-being indicators, was most prominent during training sessions in Portugal and Romania. In contrast, a notable proportion of Spanish coaches abstained from employing such tools. The preferred methodology for monitoring remained rooted in perceptual scales, while Romanian coaches indicated the use of heart rate monitors as an adjunct approach.

The following discussion delves into two key aspects of our study results: (i) a comparison of the periodic assessments and monitoring practices used in sports with the existing literature, and (ii) whether coaches are utilizing periodic assessment tests and monitoring tools that are established as both valid and reliable.

### Periodic Assessments

#### Are coaches aligning with literature suggestions in their implementation of periodic assessment practices?

Considering the current practices of physical fitness testing in sports, a recent study surveyed fifty-two sports scientists from professional soccer leagues across 18 countries [23]. The authors revealed that 58% of sports scientists and coaches incorporated physical fitness assessments during the pre-season, 42% during the in-season, and 4% during the off-season [23]. Another study that surveyed 156 strength and conditioning coaches (SCCs) from different sports, countries, and expertise levels, showed that 94% of the coaches conducted periodic assessments [24]. Predominant testing times were year-round (54%), pre-season (46%), off-season (30%), inseason (18%), and training camp (10%) [24].

These findings align with our survey, where the majority of coaches emphasize pre-season player assessments, ranging from 60.5% in Portugal to 87.7% in Romania. Conversely, in Portugal, only 26.3% assess players during the competitive season, while Romania and Spain are at 16.9% and 45.2%. A smaller portion of coaches in Portugal and Spain (13.2% and 14.5% respectively) abstain from player assessments. Romania’s non-assessment rate is even lower, at 4.6%. These findings highlight varied player assessment methods across countries. Some coaches focus on pre-season assessments, while others assess throughout the season [6, 25]. Consistent player assessment is essential for effective coaching, enabling the identification of strengths, tracking development, and adjusting strategies.

Among those who implemented physical fitness tests throughout the sports season, it was previously shown that the majority of coaches working in professional sports contexts from different modalities, included cardiorespiratory tests (92%), strength tests (81%), power tests (62%), linear speed tests (81%), acceleration tests (56%), change of direction (COD) tests (40%), and anaerobic capacity tests (31%) [23]. Our survey results revealed a similar trend at a lower extent in performance test preferences among coaches from the included countries. For instance, Portugal and Spain favored aerobic tests (50% and 46.8% respectively), while Romania leaned towards Sprint (56.9%) and skill tests (52.3%). COD tests were least used in all countries, ranging from 10.5% (Portugal) to 21% (Spain) and 18.5% (Romania). These findings suggest varied strategies for assessing athlete performance based on coaches’ preferences. Using diverse tests provides a holistic insight into athlete aptitudes. Coaches could benefit from exploring various testing methods for a comprehensive assessment.

#### What post-assessment measures do coaches utilize for training programming?

In the present study, Portuguese coaches used maximal heart rate (31.6%) through field-based tests, Spanish employed the 220-age formula to estimate the maximal HR, or perceived exertion methods (27.4%), and Romanian coaches favored training HR (26.9%) by subtracting the HR from the recovery HR, to estimate exercise intensity. A recent systematic review that analyzed studies conducted on both professional and amateur sports levels, stated that the HR measure was a common metric in the majority of the reviewed studies [14], as it serves various purposes such as indicating early signs of illness or assessing aerobic fitness. However, only two studies provided information regarding resting HR and heart rate variability (HRV) [14]. Moreover, coaches often gathered HR data more frequently than the recommended guidelines, often collecting it every session or daily [26]. However, there is a lack of information regarding the timing and frequency of data analysis, as well as whether it influenced subsequent training plans or recovery strategies [14].

Another survey conducted among SSCs who worked in Division 1 (53.1%), division 2 (28.6%), division 3 (6.1%), and division 4 (12.2%) of the Brazilian National Championship, revealed that movement velocity-based (24%) and subjective estimations (24%) are the most common, with the rating of perceived exertion (18%) and athlete-dependent approaches (16%) following [16]. Notably, in that study, only 10% of SCCs used repetition maximum (1RM) tests. Other approaches included trial and error (8%), training to failure (6%), and alternative methods (16%) [16]. These results underscore diverse SCC practices and contextual influences. In our study, Portuguese coaches favored direct (34.2%) or estimated (31.6%) maximal repetition for strength training intensity. Spanish (38.7%) and Romanian coaches (35.4%) had majorities not using intensity measures for strength training. There is an increasing interest in using velocity-based instruments, such as linear transducers, to estimate the 1RM and for training programming [27, 28]. The velocity-based training (VBT) has been preferred by coaches over traditional methods, as it allows coaches to adjust the intensity of training in real-time to align with the intended session goals [29]. This is helpful as personal 1RM can change due to factors like fatigue, nutrition, and sleep [29]. These results underscore that there is significant heterogeneity between SCCs practices, which may be due to the different sporting contexts.

For speed training programming, in the present study, the Portuguese and Romanian coaches preferred maximal speed sprint (50% and 43.1% respectively), whereas Spanish coaches lacked individualized measures (37.1%). Another previously mentioned study observed a similar trend for speed programming [16]. That study showed that maximum speed sprinting (76%), and strength outcomes (67%), are the primary measure used by coaches for enhancing speed performance [16]. The findings highlight distinct preferences among coaches from different countries for speed training programming and emphasize the importance of tailoring speed training strategies to specific national contexts and underscore the significance of incorporating maximal speed sprinting and strength training for effective speed enhancement.

### Monitoring Practices

#### Are coaches aligning with literature suggestions in their implementation of monitoring practices?

Regarding the monitoring practices, based on the survey results, there were differences in the use of training load or well-being monitoring among coaches across the different countries included in the study. Specifically, the majority of coaches in Spain (59.7%) reported not using training load or well-being monitoring in either training or competition. In Portugal and Romania, however, the majority of coaches reported using training load or well-being monitoring, but only in training sessions. The percentages were 39.5% in Portugal and 52.3% in Romania. It has been demonstrated that studies conducted at both professional and amateur sports levels commonly employ a variety of tools and measures to assess various aspects of training and performance [14]. GPS measures are employed to quantify external training load, while HR measures are utilized to gauge physiological. On the other hand, different RPE scales are commonly used to evaluate the physiological response to training sessions, and self-report questionnaires were employed to assess the psychological responses to training [14].

In our study, of the coaches utilizing training monitoring tools to regulate training intensity, a significant proportion indicated reliance on heart rate, with prevalence rates of 28.9% in Portugal and 32.3% in Romania. Furthermore, both Portuguese and Spanish coaches incorporated the rate of perceived exertion as a monitoring metric, accounting for 26.3% in Portugal and 21% in Spain. Conversely, in Romania, a relatively smaller proportion, specifically 12.3% of coaches, reported utilizing such perceptual scales for monitoring. Although in the last decade, there has been a growing interest in the investigation of GPS systems for quantifying training intensity [12, 30], our study showed that the majority of the coaches from the three countries opted to use RPE scales and HR monitors. This can be explained by the fact that GPS systems, despite being more accessible in terms of cost, the majority of the clubs cannot afford such instruments for training intensity monitoring.

Considering the well-being/recovery monitoring practices, the majority of coaches in Portugal (57.9%), Spain (53.2%), and Romania (44.6%) reported using well-being perceptive scales to monitor recovery status. In addition to well-being perceptive scales, the second most used instrument for monitoring recovery status among coaches in Portugal and Romania was heart rate variability, with percentages of 31.6% and 33.8%, respectively. In Spain, the percentage of coaches using heart rate variability was higher, with a percentage of 41.9%. Contrary to our results, a systematic review showed that the utilization of well-being/recovery surveys was limited [14]. Specifically, within the mentioned investigation [14], only 13% of participants employed the recovery stress questionnaire for athletes. In comparison, both the profile of mood states (POMS) and the daily analysis of life demand a mere 2% of employed athletes (DALDA).

Overall, these findings suggest that there may be a lack of consistency in the use of monitoring tools for controlling training intensity among coaches across different countries and contexts. Still, this holistic approach indicates that a combination of objective and subjective measures was employed to comprehensively understand athletes’ responses to training and their overall performance. Also, there may be differences in the use of instruments/measures for monitoring recovery status among coaches across different countries and contexts.

#### Are coaches harnessing periodic assessment tests and monitoring tools that demonstrate validity and reliability?

The surveyed coaches in the present study used field-based tests and the 220-age formula to estimate maximal heart rate. Considering strength tests, coaches favored direct and estimated 1RM methods. Whereas for speed measures, coaches preferred using maximal speed sprint measure.

Coaches play a pivotal role in optimizing athletes’ training programs through informed decision-making. The insights garnered from this study’s findings underscore the significance of utilizing periodic assessment tests and monitoring tools that possess proven validity and reliability. Particularly, the reliance on field-based tests and the 220-age formula for estimating maximal heart rate, as observed among the surveyed coaches, aligns with the established validity and reliability of these methods [31, 32]. The fact that the age-predicted HR_max_ equations did not exhibit comparable levels of validity and reliability in predicting HR_max_ implies the need for caution when employing such equations for training prescription. Similarly, coaches’ inclination towards both direct and estimated 1RM methods for strength assessments is warranted by the robust test-retest reliability demonstrated across various factors, thereby ensuring accurate strength profiling [33]. In terms of speed measures, the preference for maximal speed sprint tests finds support in their recognized validity and reliability for programming effective speed training sessions [34].

Furthermore, the use of the rate of perceived exertion scales to monitor training intensity is one of the most common methods used by coaches [14]. In fact, the RPE method was proved to be both valid and reliable in its origins, with the RPE demonstrating a strong relationship with HR and work intensity [35]. With the evolution of the RPE measure in recent decades, other metrics, such as session-RPE (which relates to the overall session intensity rather than focusing on individual exercises), have also proven to be valid and reliable measures of intensity. [36].

Considering the perceptual scales of well-being and recovery status, its use is a crucial aspect that should be incorporated alongside performance enhancement, especially in elite-level sports that help limit the presence of bad overreaching and/or overtraining syndrome [37, 38]. Moreover, diverse methodologies are employed for the application and analysis of single-item self-report measures in the monitoring of team-sport athletes [39]. Both composite and single-item wellness measures have demonstrated diverse associations with training load measures, spanning from negligible correlations to substantial relationships [39]. Subjective well-being consistently reflects the impact of training stress, declining with heightened and prolonged training, and rebounding with reduced training [21]. Given their responsiveness in reflecting changes in athlete well-being, coaches can confidently utilize self-report measures for athlete wellbeing and recovery monitoring.

Coaches should prioritize evidence-based assessment tools and techniques that have been proven to be both valid and reliable [40]. This approach ensures that the information gathered from assessments accurately reflects athletes’ physiological capacities and helps guide personalized training interventions. By making informed decisions based on reliable data, coaches can optimize training programming, monitor progress, and ultimately enhance athletes’ overall performance outcomes [18]. The fact that coaches from different countries are utilizing these techniques is indicative of a growing awareness within the coaching community about the importance of valid and reliable assessment methods. This convergence in practices suggests a positive trend toward evidence-based coaching approaches.

### Limitations

While this study provides valuable insights into the practices of sports coaches in Portugal, Spain, and Romania regarding periodic assessments and monitoring tools, it is important to acknowledge certain limitations that may influence the interpretation of the findings. Firstly, the study’s sample size and geographical scope may limit the generalizability of the results to a broader international context. The surveyed coaches were exclusively from these three European countries, and their practices might not fully represent those of coaches from other regions with distinct sporting cultures and infrastructures. Therefore, caution should be exercised when extrapolating these findings to a global coaching population.

Secondly, the utilization of self-report data through the survey method introduces the potential for response bias. Coaches’ perceptions and self-reported practices may be influenced by memory recall, social desirability, and other cognitive factors that could impact the accuracy and completeness of the information provided. Despite efforts to ensure anonymity and confidentiality, it is impossible to entirely eliminate the influence of these biases. Moreover, the survey questionnaire was crafted based on existing literature and the research objectives. The study primarily relied on quantitative data to analyze coaches’ practices, offering a limited exploration of the underlying reasons and contexts for their choices. Lastly, the study centered on the practices of coaches themselves, without directly evaluating the effectiveness or impact of these practices on athlete performance or well-being. While the study provides insights into coaches’ behaviors, future research could delve into the outcomes of these practices on athletes’ performance, injury prevention, and overall well-being.

## CONCLUSIONS

The findings of the present study underscore notable distinctions in assessment protocols and the adoption of training load/well-being monitoring tools across coaches in Portugal, Spain, and Romania. The prospects of embracing diverse methodologies for gauging intensity and overseeing recovery merits careful consideration, potentially yielding synergistic insights that culminate in a more comprehensive grasp of athlete performance dynamics. These insights furnish a robust framework for curating bespoke educational resources that cater to the unique exigencies of coaches, thereby engendering a substantive advancement in the realm of sports coaching.

## Ethical approval

The study received approval from the Ethics Committee for Social Sciences, Life, and Health at Instituto Politécnico de Viana do Castelo, under reference number 4/A/2023.
